# MDC1 methylation mediated by lysine methyltransferases EHMT1 and EHMT2 regulates active ATM accumulation flanking DNA damage sites

**DOI:** 10.1038/s41598-018-29239-3

**Published:** 2018-07-18

**Authors:** Sugiko Watanabe, Makoto Iimori, David Virya Chan, Eiji Hara, Hiroyuki Kitao, Yoshihiko Maehara

**Affiliations:** 1 Innovative Anticancer Strategy for Therapeutics and Diagnosis Group, Innovation Center for Medical Redox Navigation, Kyushu University, Fukuoka, 812-8582 Japan; 2 Department of Molecular Microbiology, Research Institute for Microbial Diseases, Osaka University, Suita, Osaka 565-0871 Japan; 3 Department of Molecular Cancer Biology, Graduate school of Pharmaceutical Sciences, Kyushu University, Fukuoka, 812-8582 Japan; 4 Department of Surgery and Science, Graduate school of Medical Sciences, Kyushu University, Fukuoka, 812-8582 Japan

## Abstract

Chromatin dynamics mediated by post-translational modifications play a crucial role in cellular response to genotoxic stress for the maintenance of genome integrity. MDC1 is a pivotal chromatin adaptor in DNA damage response (DDR) and its methylation is essential to recruit repair factors at DNA double-strand break (DSB) sites, yet their precise molecular mechanisms remain elusive. Here we identified euchromatic histone-lysine *N*-methyltransferase 1 (EHMT1) and EHMT2 as novel regulators of MDC1, which is required for the accumulation of DDR factors e.g. 53BP1 and RAP80, at the DSB sites. MDC1 interacts mainly with EHMT1, which is facilitated by DNA damage-initiated ATM signalling, and EHMT2 dominantly modulates methylation of MDC1 lysine 45. This regulatory modification promotes the interaction between MDC1 and ATM to expand activated ATM on damaged chromatin and dysfunctional telomere. These findings identify EHMT1 and EHMT2 as DDR components, with implications for genome-integrity maintenance through proper dynamic methylation of MDC1.

## Introduction

Cells are constantly assaulted by intrinsic (e.g. reactive oxygen species) and environmental (e.g. ultraviolet light, ionizing radiation, chemical agents) factors that can induce genotoxic insults^1,2^. To counteract the threats to genome integrity, cells have evolved a sophisticated mechanisms of DNA damage responses (DDR) and associated downstream repair pathways. In this process, complex networks of multiple proteins are tightly controlled by posttranslational modifications and protein-protein interactions, facilitating the signal propagation and DNA repair^1–5^.

One of the most harmful form of DNA damage is the double-strand breaks (DSBs), and the mediator of DNA damage checkpoint 1 (MDC1) is a central player in the DDR to DSBs. MDC1 binds directly to γ-H2AX and recruits the Mre11-Rad50-Nbs1 (MRN) complex, ATM and many additional downstream mediators to DNA break sites, leading to the amplification of DDR signal, coordinating checkpoint activation, apoptosis and DSB repair. Beyond its contribution to DDR, the precise mechanisms of MDC1 regulation remain largely unknown. MDC1 is subjected to several post-translational modifications, including phosphorylation, ubiquitylation and methylation, and its modification cascade or dynamics is important for the recruitment of repair mediators, such as 53BP1 and RAP80/BRCA1 complex, in the DDR. Among them, the methylation of MDC1 at lysine 45 could be essential for DNA damage-induced recruitment of repair factors to DSBs^6^.

In this study, among the candidate lysine methyltransferases regulating MDC1 in DDR, EHMT1 [also known as G9A-like protein; GLP or KMT1D (lysine methyltransferase 1D)] and EHMT2 [also known as G9A or KMT1C (lysine methyltransferase 1C)] were identified as mediators for the proper methylation of MDC1. Interestingly, recent studies have shown that EHMT2^7^ and its catalytic activity^8,9^ promote DNA repair in response to DSB-inducing genotoxic stress. However, the precise mechanism of EHMT2 linked to EHMT1 in DDR remain elusive. Here, we show that EHMT1 and EHMT2-dependent methylation of MDC1 is required for the interaction between activated ATM and MDC1 to amplify the DDR signal, followed by recruitment of repair factors to DNA damage sites including the dysfunctional telomere. These data enhance our understanding of EHMT1 and EHMT2 as DDR components, through proper dynamic methylation of MDC1 to maintain genome integrity.

## Results

### EHMT1 and EHMT2 are required for recruitment of MDC1-downstream factors to DNA damage sites

In an attempt to identify a potential lysine methyltransferase (KMT) targeting MDC1 within the DDR, we monitored the accumulation of its downstream factors in the DNA damage-induced foci in U2OS cells with small interfering (si)RNA-mediated knockdown of KMTs. We treated cells with neocarzinostatin (NCS), a radiomimetic DNA damage agent, and observed the ubiquitin conjugates immunodetected by the anti-ubiquitin antibody FK2 and the accumulation of 53BP1 at the DNA damage sites (Fig. S1a,b). Among the 9 enzymes tested, the depletion of EHMT1, EHMT2 and SETD8, but not SUV39H1, SETDB1 and MLL1-4, impaired the accumulation of ubiquitin conjugates and 53BP1. When SETD8 was depleted, the distribution of MDC1 was perturbed even in unstressed conditions and impaired the accumulation of MDC1 and ubiquitin conjugates in response to DNA damages (Fig. S2b,c). Supportively, several reports have shown that the depletion of SETD8 leads to genomic instability even under unperturbed conditions^10,11^, and that SETD8 is recruited to DSBs and required for NHEJ-directed repair^12,13^. These observations raise the possibility that SETD8 might adjust the physiological structure of chromatin as a fundamental regulator to protect against genomic insults. In contrast, depletion of EHMT1 or EHMT2 had little effect on MDC1 recruitment to damaged chromatin, but it had markedly reduced the accumulation of downstream factors of MDC1 in DDR (Figs S1a and S2a). Therefore, we focused on these two methyltransferases, EHMT1 and EHMT2, which form a heterodimeric complex, as candidates for regulators of MDC1.

**Figure 1:**
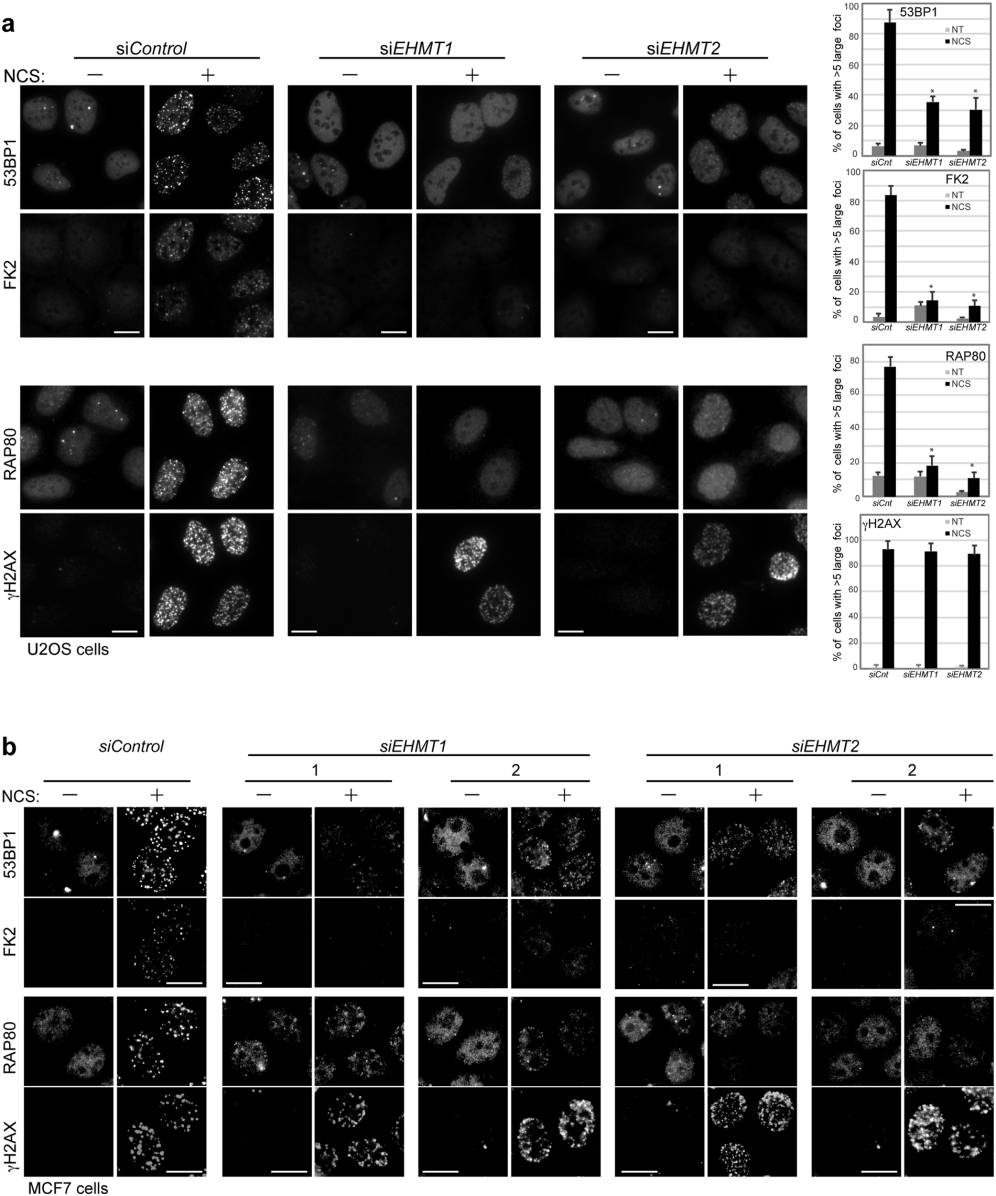
EHMT1 and EHMT2 are required for accumulation of ubiquitin conjugates and repair factors at DNA damage sites. (**a**,**b**) Immunofluorescence analysis of U2OS cells (**a**) and MCF7 (**b**) cells transfected with indicated siRNA, and co-immunostained with indicated antibodies at 2 h after exposure to neocarzinostatin (NCS, 50 ng/ml for 15 min). A representative image of each treated or control cells is shown, as indicated. DNA damage induced foci are quantified as the percentage of cells with more than 5 large foci in nuclei after background subtraction, each based on at least 150 cells from three independent experiments (right). Error bars represent standard deviation (SD). Statistical significance was calculated using two-tailed, unpaired t-test compared with control cells; *P < 0.0001. The knockdown efficiencies with individual siRNAs against EHMT1 and EHMT2 are shown in Fig. S3a,b. Scale bar, 10 μm.

To investigate whether EHMT1 and EHMT2 affects MDC1 function as a platform for integrating in or operating the DSB-triggered pathway, we observed local ubiquitylation and subsequent recruitment of repair factors, RAP80 and 53BP1, at the site of damaged chromatin using immunofluorescent analysis. We treated cells with NCS and found that depletion of EHMT1 and/or EHMT2 impaired the accumulation of conjugated ubiquitin, 53BP1 and RAP80 at the DSB sites in all three cell types examined, U2OS, MCF7 and diploid human lung fibroblast cells, using multiple siRNAs targeting *EHMT*1 or *EHMT*2 (Figs 1a,b and S3). These results suggest that EHMT1 and EHMT2 are required for the efficient formation of ubiquitin conjugates followed by the recruitment of repair factors at the DNA-damage sites.

### EHMT1 interacts with MDC1 and is facilitated by ATM activation in DDR

**Figure 2:**
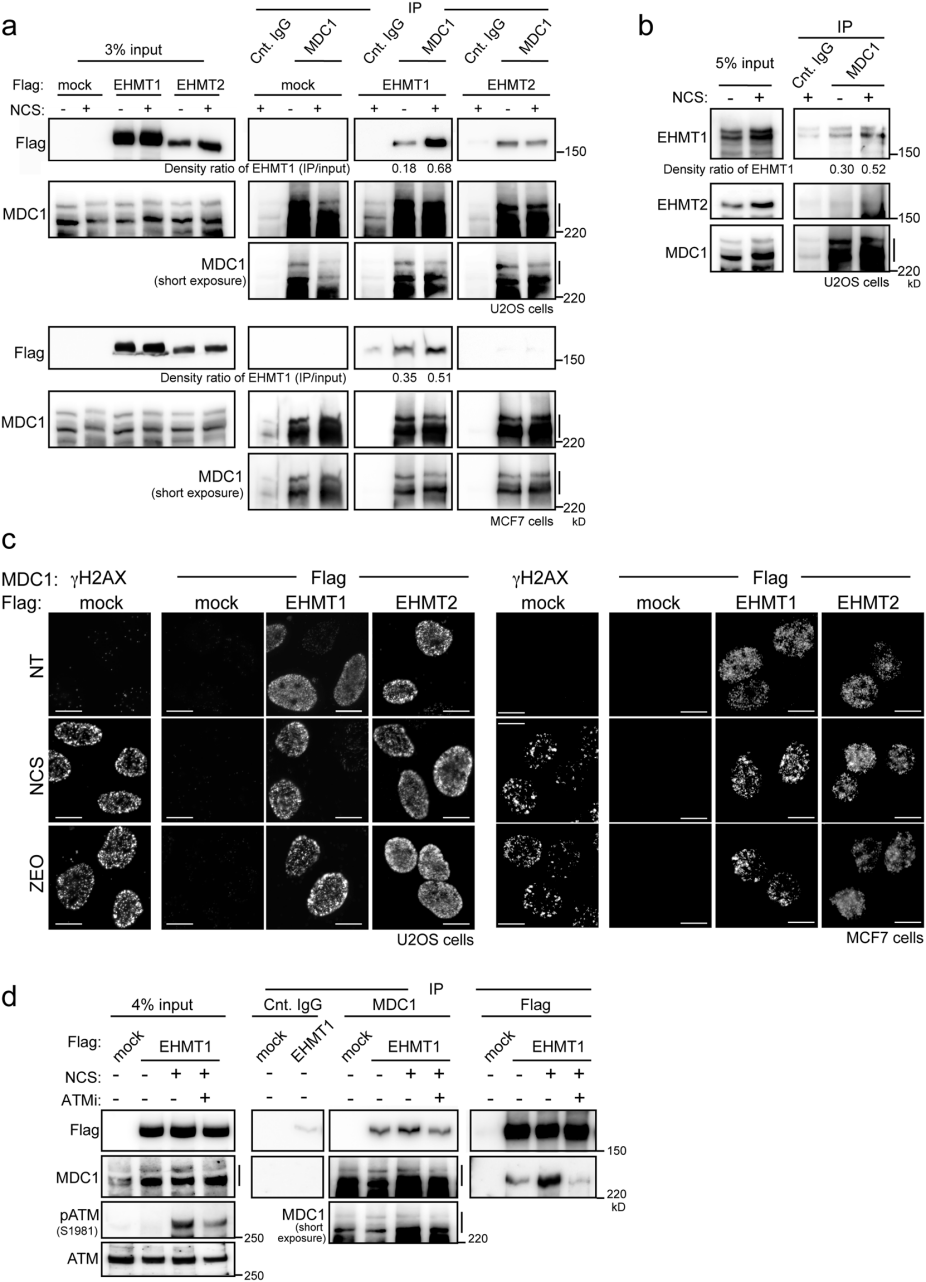
MDC1 interacts with EHMT1/EHMT2 in DNA damage response. (**a**,**b**) Immunoprecipitation of endogenous MDC1 (**a**) with FLAG-tagged EHMT1 or EHMT2 in U2OS and MCF7 cells, or (**b**) with endogenous EHMT1 or EHMT2 in U2OS cells, exposed or not to NCS (500 ng/ml for 30 min) as indicated. Density ratio of EHMT1 (IP/input) was quantified and indicated at the bottom of panel. (**c**) *In situ* proximity ligation assay of U2OS cells (left panels) and MCF7 cells (right panels) transfected with FLAG-tagged mock, EHMT1 or EHMT2, subjected to immunoreaction using MDC1 antibodies combined with γH2AX or Flag antibodies at 1 h after exposure to NCS (50 ng/ml for 15 min) or Zeocin (ZEO 100 μg/ml for 1 h). Scale bar, 10 μm. (**d**) Immunoprecipitation of endogenous MDC1 and FLAG-tagged EHMT1 in U2OS cells treated with 10 μM of ATM inhibitor for 100 minutes followed by exposure of NCS (500 ng/ml for 30 min). Line marking the multiple bands of MDC1 apparently represent alternatively spliced forms^20^ (**a**,**b** and **d**).

To determine whether EHMT1/EHMT2 lysine methyltransferases physically interact with MDC1, lysates of U2OS and MCF7 cells expressing Flag-tagged EHMT1 or EHMT2 were prepared and immunoprecipitation analysis was performed upon the treatment of NCS. We found that the interactions between MDC1 and EHMT1 were detectable even under undamaged conditions, and enhanced by treatment with NCS in these cell lines (Fig. 2a). On the other hand, EHMT2 marginally associated with MDC1 only in U2OS cells, and their interaction was not changed by treatment with NCS. By employing immunoprecipitation of untransfected cells, we confirmed that the endogenous MDC1 interacts with EHMT1, but only a modest MDC1-EHMT2 association was detected (Fig. 2b). From a morphological approach, the *in situ* proximity ligation assay (PLA) enabled the visualization of the colocalization of MDC1 with EHMT1 or EHMT2 upon exposure to radiomimetic drugs (NCS or Zeocin), in combination with Flag antibody detecting EHMT1 or EHMT2 and MDC1 antibody (Fig. 2c)^14^. The PLA signals of MDC1-EHMT1 and MDC1-EHMT2 were detectable as fine intranuclear foci even under undamaged conditions. Subsequent to DNA damage, the EHMT1-MDC1 PLA signals exhibited a focal accumulation pattern in the nuclei, but EHMT2-MDC1 PLA signals did not change their distributions (Fig. 2c). These results imply that EHMT1 exerts a proactive role in MDC1 regulation by responding to signals induced by DNA damage. To test this notion, we examined whether the ATM activity affects the enhanced interaction of EHMT1 with MDC1 upon DNA damage. As expected, the immunoprecipitation analysis revealed that inhibition of ATM kinase activity by treatment with KU-55933 abolished the increased EHMT1-MDC1 interaction upon exposure to NCS (Fig. 2d). These results indicate that MDC1 interacts mainly with EHMT1 in the absence of DNA damage, and their interaction is facilitated by the DNA damage-initiated ATM signalling.

### EHMT2 promotes methylation of MDC1 lysine 45

**Figure 3:**
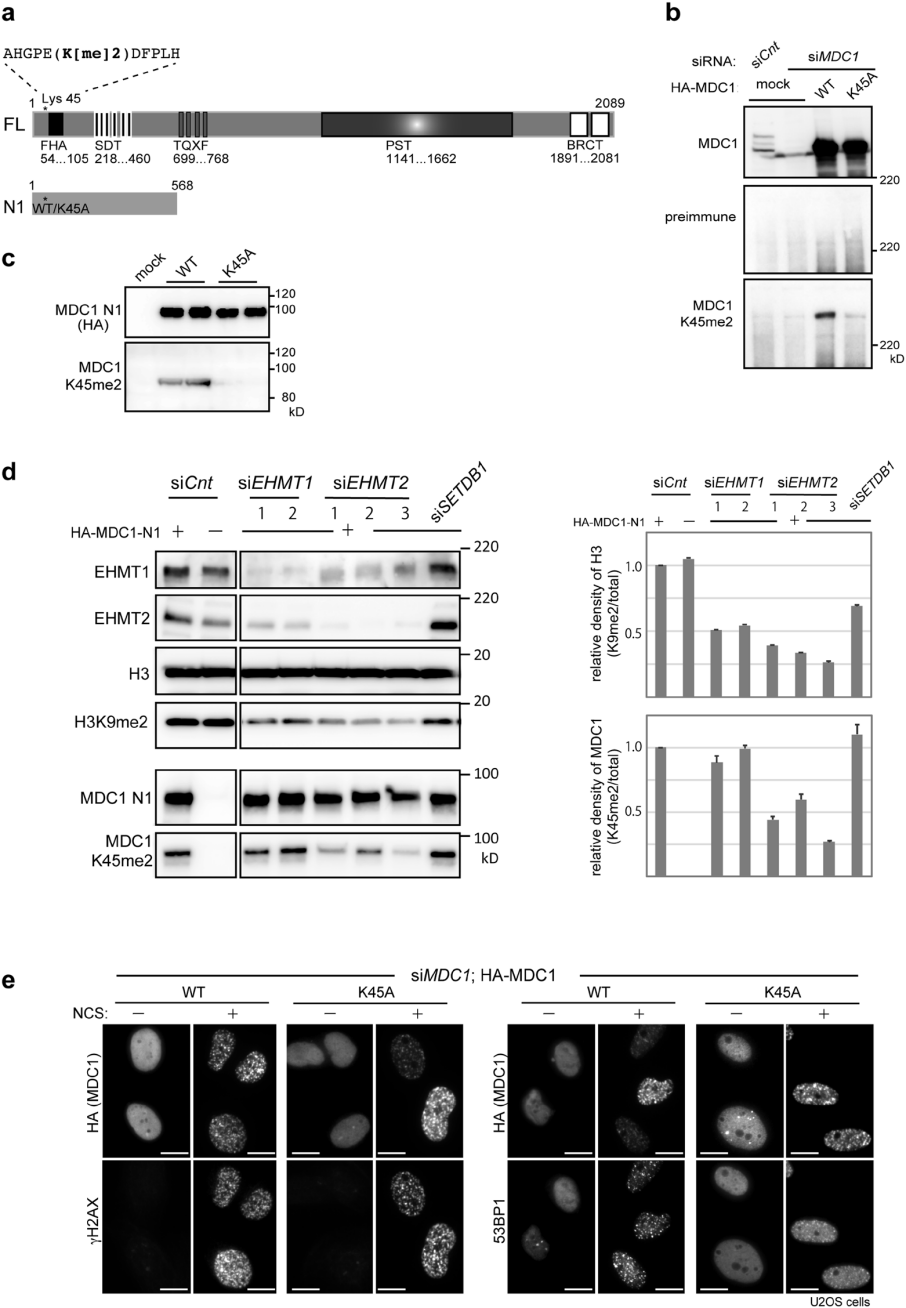
EHMT2 promote methylation of MDC1 at lysine 45. (**a**) Schematic illustration of the full-length (FL) and deletion mutant (N1) of MDC1. Asterisk indicates the position of lysine 45 (wild type; WT or the alanine-substituted mutant; K45A). FHA, forkhead-associated domain; SDT, Ser-Asp-Thr repeats; TQXF, Thr-Gln-X-Phe repeats; PST, Pro-Ser-Thr repeats; BRCT, tandem BRCT domain. (**b–d**) Immunoblotting analysis of whole-cell extracts from U2OS cells transfected with (**b**) HA-tagged MDC1 (WT or K45A) and control or *MDC1*-targeting siRNAs, (**c**) HA-tagged MDC1-N1 (WT or K45A), and (**d**) HA-tagged MDC1-N1 (WT) and control, two *EHMT1*-targeting siRNAs (*EHMT1* siRNA-1 and siRNA-2) or three *EHMT2*-targeting siRNAs (*EHMT1* siRNA-1 siRNA-2 and siRNA-3; see methods), using the indicated antibodies. Quantification of methylation levels in histone H3 and MDC1 shown in right panels. Relative density was calculated based on signal intensities normalized against levels of total histone H3 (upper panel) and total MDC1-N1 (lower panel). Error bars, SD from triplicate measurements. (**e**) Immunofluorescence analysis of U2OS cells transfected with HA-tagged MDC1 (WT or K45A) and *MDC1*-targeting siRNA, and co-immunostained with indicated antibodies at 2 h after exposure to neocarzinostatin (NCS, 50 ng/ml for 15 min). A representative image of each treated or control cells is shown, as indicated. Scale bar, 10 μm.

To assess whether EHMT1 and EHMT2 are responsible for the methylation status of MDC1, we raised an antiserum against the dimethylated lysine 45 by using a synthetic methylated peptide as an antigen (Fig. 3a). Our results from the immunoblotting experiments shown in Fig. 3b revealed that the antibody specifically recognized the WT-MDC1 only, but not the unmethylated mutant, K45A-MDC1^6^, in comparison with the pan-MDC1 (Fig. 3b; upper) and the preimmune control (Fig. 3b; middle) antibodies.

To investigate the effects of EHMT1 and EHMT2 on the methylation levels of MDC1, we generated a N-terminal variant of MDC1 containing lysine 45, whose methylation could be detected with a methyl-specific antibody (Fig. 3c), cotransfected with siRNA against *EHMT*1 and *EHMT*2 into U2OS cells, and the cell lysates were subjected to immunoblotting experiments (Fig. 3d). As previously reported^15,16^, the levels of H3K9me2 were decreased in either EHMT1 or EHMT2 depleted cells (Fig. 3d; right). On the other hand, depletion of EHMT2, but not of EHMT1, reduced the methylation levels of MDC1, indicating that EHMT2 is required for modifying MDC1 methylation. Consistently, the catalytic activity of EHMT1 but not EHMT2 is dispensable for their methyltransferase activity *in vivo*^17^, while the methylation-binding activity of EHMT1 in EHMT1/EHMT2 heteromeric formation is essential for gene silencing^18,19^. These observations imply that EHMT1 and EHMT2 have distinct roles, for example, a regulating function through its interactor and catalytic activity, respectively (see Discussion). However, to clarify whether these molecules act through a heterodimeric complex or could function in an independent manner during the DDR process requires further investigation.

### Methylation of MDC1 lysine 45 is required for ATM accumulation on damage sites

**Figure 4:**
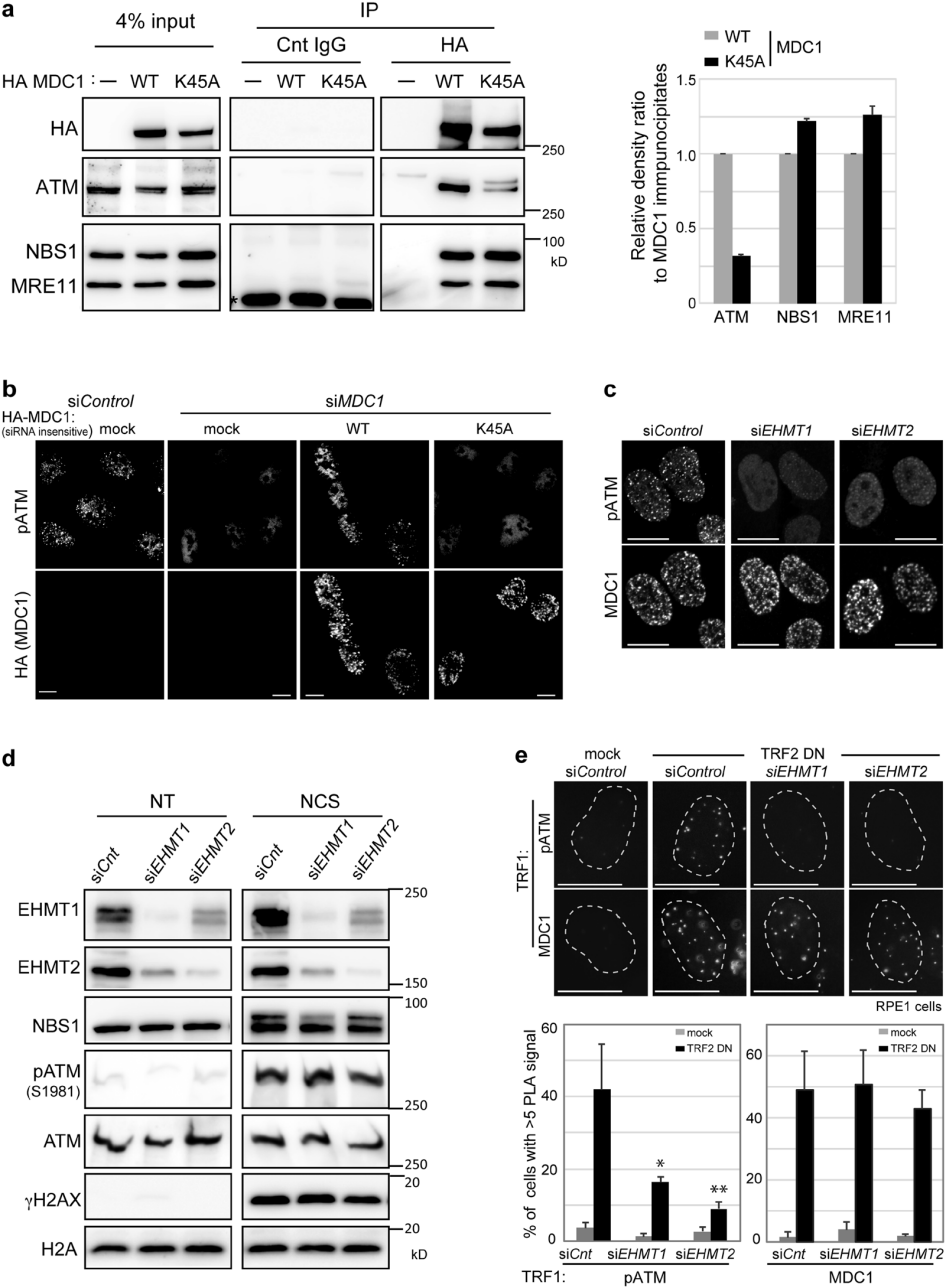
MDC1 methylation is required for the amplification of activated ATM around the DNA double strand break sites. (**a**) Coimmunoprecipitation of MDC1 with ATM, NBS1 or MRE11 from U2OS cells transfected with HA-tagged MDC1 (WT or K45A). Asterisk: non-specific bands. Right panels: Quantification of immunoprecipitated ATM, NBS1 and MRE11 calculated from the signal intensities normalized against level of MDC1 immunoprecipitates. Error bars, SD from triplicate measurements. (**b**,**c**) Immunofluorescence analysis of U2OS cells transfected with indicated siRNA oligonucleotides, (**b**) and siRNA-resistant HA-tagged MDC1 (WT or K45A), using indicated antibodies at 2 h after exposure to NCS (50 ng/ml for 15 min). Scale bar, 10 μm. (**d**) Immunoblotting analysis of whole-cell extracts from U2OS cells transfected with control, *EHMT1*- or *EHMT2*-targeting siRNAs, using the indicated antibodies at 2 h after exposure to NCS (100 ng/ml for 30 min). (**e**) *In situ* PLA of RPE1 cells introduced with mock or TRF2-DN combined with or without the knockdown of EHMT1 or EHMT2, subjected to immunoreaction using TRF1 antibodies combined with MDC1 or phosphor-ser1981 ATM antibodies. Representative images are shown in the left panel. Localization of MDC1 or activated ATM at dysfunctional telomeres are quantified as the percentage of cells with more than 5 PLA signals in nuclei, each based on at least 150 cells from three independent experiments (right). Error bars represent SD. Statistical significance was calculated using two-tailed, unpaired t-test compared with control cells introduced with TRF2-DN; *P < 0.0005, **P < 0.0001. Scale bar, 10 μm.

Protein methylation frequently mediates protein-protein interaction transducing to downstream biological outcomes. In fact, proper dynamic methylation of MDC1 is essential for assembling the DDR factors, including ubiquitin ligase complexes and repair mediators^6^, although the constitutively unmethylated MDC1 K45A variant itself is still accumulated around the sites of DNA damage (Fig. 3e). Furthermore, MDC1 serves as a platform to iterate the MRN complex-ATM activation loop, thus propagating the DDR signal around the damage sites^5^. Since the FHA domain of MDC1, which is located near the methylation lysine 45 on MDC1 (Fig. 3a), interacts with the MRN complex^20,21^ and ATM^22^, it is conceivable that their interaction might be regulated by MDC1 methylation. To test this hypothesis, we performed immunoprecipitation using cells expressing either the MDC1-wild type or unmethylated mutant MDC1-K45A. NBS1 and MRE11 interacted with both the wild type and the unmethylated mutant of MDC1, while ATM coimmunoprecipitated with the wild-type MDC1, but less robustly with K45A (Fig. 4a). Consistent with these results, immunofluorescence analysis also revealed that the accumulation of activated ATM around the damaged sites was impaired by depletion of endogenous MDC1 and was restored by expression of siRNA-resistant sequence encoding HA-tagged wild type MDC1, but not by the MDC1 K45A variant (Fig. 4b). Furthermore, depletion of EHMT1 or EHMT2 abolished the accumulation of activated ATM in response to DNA damage (Fig. 4c), while the total amount of autophosphorylated ATM remained unaffected (Fig. 4d). A previous report supporting this observation showed that the activated ATM failed to accumulate near DSBs in MDC1^−/−^ cells, even though immunostaining signals of the phospho-ATM were increased upon DNA damage, and that MDC1 with a deleted FHA domain in MDC1^−/−^ cells failed to restore the phospho-ATM foci formation despite itself forming the MDC1 foci following DNA damage^22^. Collectively, these data suggest that methylation of MDC1 mediated by EHMT1 and EHMT2 is required for the interaction between ATM and MDC1, facilitating the accumulation of activated ATM at the DSB sites, and could conceivably regulate the FHA domain of MDC1.

Furthermore, on uncapped telomeres, ATR/ATM kinases and repair factors including MDC1 are known to be activated and form telomere dysfunction-induced foci (TIFs) in the nuclei^23^. Reportedly, MDC1 promotes the frequency of cells with phosphorylated ATM foci at uncapped telomere lesions, as it does at DSBs^24^. In line with these observations, we examined whether methylation of MDC1 affects the accumulation of activated ATM also at dysfunctional telomeres. Telomere dysfunction was induced by retrovirus introduction of dominant negative TRF2 (TRF2-DN)^23,24^ in RPE-1 cells, a human telomerase-immortalized retinal pigment epithelial line, with or without the knockdown of EHMT1 or EHMT2. The TIFs were scored by PLA between MDC1 or activated ATM with another shelterin component TRF1, which is known to be at the telomere dysfunction lesions even in the absence of TRF2 (Fig. 4e). MDC1 itself could localize to the telomere dysfunction lesions irrespective of the presence or absence of these methyltransferases. In contrast, activated ATM did not accumulate at telomere dysfunction lesions in EHMT1- or EHMT2-depleted cells. These data indicate that EHMT1 and EHMT2 are required for the accumulation of activated ATM, but not of MDC1, at telomere dysfunction lesions.

In conclusion, the EHMT1 and EHMT2 interact with MDC1, and interaction between EHMT1-MDC1 is facilitated by ATM activation in DDR. Methylation of MDC1 lysine 45 is modulated mainly by EHMT2 and this methylation is required for the accumulation of activated ATM around the DNA damage sites, including the dysfunctional telomere.

## Discussion

**Figure 5:**
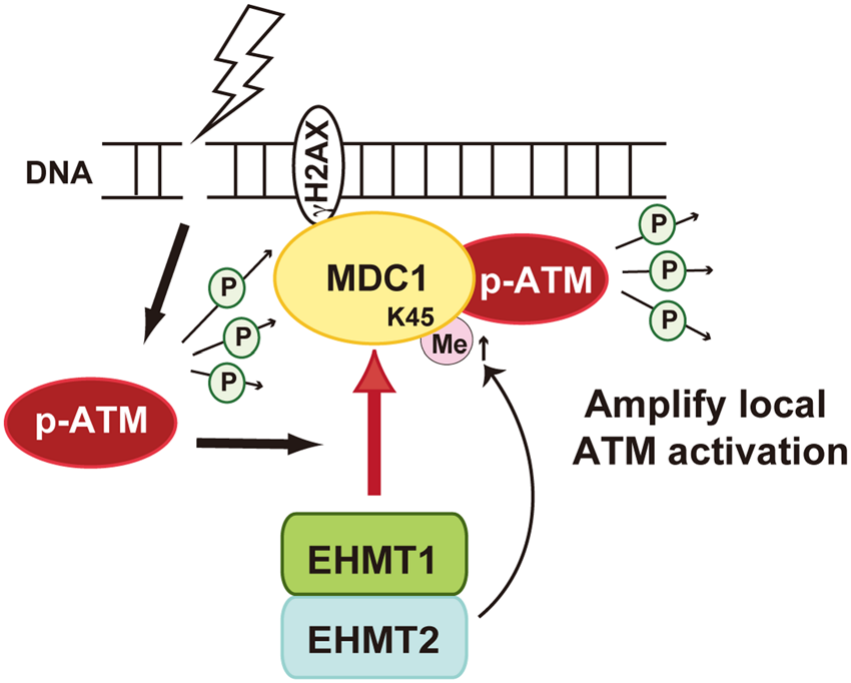
Schematic model of EHMT1/EHMT2 heterodimer-mediated accumulation of activated ATM to DSBs.

Our results suggest the following scenario. EHMT1 is recruited to MDC1 on damaged chromatin in an ATM-dependent manner. The EHMT2 in EHMT1/EHMT2 heterodimer methylates MDC1 to amplify the local activation of ATM to expand the phosphor-signal around the damaged chromatin (Fig. 5). In our finding, unmethylated MDC1 at lysine 45 failed to amplify ATM accumulation on damaged chromatin and to recruit downstream repair factors, while ATM activation itself was observed in EHMT1- or EHMT2-depleted cells (Fig. 4). Consistent with our data, a previous report showed that activated ATM completely failed to accumulate near the DSBs in *MDC1*^−/−^ MEF^22^, even though phospho-ATM immuno- staining signals were increased following DNA damage, and that unmethylated MDC1 could form a robust non-chromatin complex with downstream repair factors^6^. Together, these findings suggest that the methylation of MDC1 at lysine 45 might have a crucial role of MDC1 as a platform for the association of activated ATM and downstream factors on the DSB-flanking chromatin.

For the accumulation of methylated MDC1 on damaged chromatin, the ATM signalling initiated by DNA damage primarily facilitates the EHMT1-MDC1 interaction (Fig. 2). In fact, several sites with an S/TQ motif in MDC1 are already reported to be phosphorylated by ATM, thereby propagating the DDR signal on damaged chromatin^4^. On the other hand, the EHMT1 also contain six S/TQ motifs conserved in human, mouse and chicken, which are candidates for ATM phosphorylation, and actually EHMT1 has been detected in a screen for ATM/ATR substrate in DDR^25^. However, individual EHMT1 mutants comprising alanine substitutions of these S/TQ motifs could interact with MDC1 in damage inducible manner (data not shown). Interestingly, a recent report has demonstrated that EHMT2 is phosphorylated by ATM and this phosphorylation is required for EHMT1 localization on damaged chromatin treated by laser micro-irradiation^9^. Possibly, phosphorylation of MDC1, hyperphosphorylation of EHMT1, or another factor mediated by ATM kinase may be important for facilitating the MDC1-EHMT1 association. Further investigation is required to understand the molecular mechanism of this system.

As the DDR components, EHMT1 and EHMT2 seem to have distinct roles. For example, EHMT1 exerts a proactive role in MDC1 regulation by responding to signals induced by DNA damage (Fig. 2), whereas EHMT2 mainly contributes to the methylation levels of MDC1 (Fig. 3). Consistently, a previous study demonstrated that the catalytically inactive EHMT1 formed a heterodimer with EHMT2 that could restore the methylation level of histone H3 lysine3 in *Ehmt1*^−/−^ MEF, while the catalytically inactive EHMT2 failed to do so in *Ehmt2*^−/−^ MEF^17^. Therefore, EHMT2, but not EHMT1, is indispensable for their methyltransferase activity. On the other hand, since methylation-binding activity of EHMT1 in EHMT1/EHMT2 heteromeric formation is essential for gene silencing^18,19^, EHMT1 could exert its biological function by interacting with targets, that is, MDC1 on damaged chromatin.

Furthermore, depletion of EHMT1 or EHMT2 abrogates the recruitment of ATM to the unprotected chromosome ends (Fig. 4e). Even if MDC1 is recruited to a dysfunctional telomere, ATM signal is not sufficiently activated on damaged chromatin without EHMT1 or EHMT2 (Fig. 4c). Given that EHMT1 and EHMT2 are degraded in senescent cells^26^, the prevention of excessive DDR through degradation of EHMT1 and EHMT2 might be required for the survival of replicative senescent cells that possess shorten telomeres. Moreover, the DDR activation on eroded telomeres followed by cell division causes genomic instability through chromosomal breakage-fusion-bridge cycles, which could develop cancer. Indeed, since EHMT2 is often overexpressed and associated with poor prognosis in several cancers^27^, the (re)activation of EHMT2 might contribute to tumorigenesis by leading chromosomal instability caused by persistent DDR.

Taken together, these findings enhance our understanding of the DDR mechanisms through proper dynamic methylation of MDC1, with implications for genome integrity.

## Methods

### Plasmids and transfection

Total RNA from MRC5 cells was isolated using an RNeasy mini kit (Qiagen) and reverse-transcribed using a SuperScript III (Invitrogen). Human EHMT1 was amplified by PCR using cDNA from MRC5 cells and cloned into the pFLAG-CMV2 or p3XFLAG-CMV7.1 vector (SIGMA). Full-length human MDC1 cDNA in pENTR was obtained from Addgene (plasmid #26427) and was subcloned into pcDNA-DEST-HA vector in our laboratory stock, using the Gateway cloning technology according to the manufacturer’s instructions (Thermo Fisher Scientific). All clone sequences were confirmed. FLAG-EHMT2 was as previously described^26^. The plasmids were transfected into the cells using Xtreme HP reagent (Roche) according to the manufacturer’s protocol.

### Cell culture and treatment

U2OS, MCF7, TIG3 and MRC5 cells (American Type Culture Collection) were grown in DMEM supplemented with 10% (v/v) heat-inactivated fetal bovine serum (Thermo Fisher Scientific). Where indicated, the culture medium was supplied by ATM inhibitor KU55933 (Sigma-SML1109: 10 μM) and neocarzinostatin (Sigma-N9162).

### Small interfering RNA (siRNA)-mediated knockdown

siRNA oligonucleotides used were as follows (sense strands): *EHMT1*-*1*: 5′-CAGCUGCAGUAUCUCGGAAtt-3′, *EHMT1*-*2*: 5′-GGACGGUGAUUGAGAUGUUtt-3′, *EHMT2*-*1*: 5′-GCUCUAACUGAACAACUAAtt-3′, *EHMT2*-*2*: 5′-CGCUGAUUUUCGAGUGUAAtt-3′, *EHMT2*-3: 5′-CCAUGAACAUCGAUCGCAAtt-3′, *GL3* for control: 5′-CUUACGCUGAGUACUUCGAtt-3′. The siRNAs were transfected into the cells using Oligofectamine RNAiMAX (Thermo Fisher Scientific) according to the manufacturer’s protocols.

### Antibodies

Used antibodies and dilution rate are indicated as follows; IF, immunofluorescence; WB, Western blotting; IP, immunoprecipitation. 53BP1 (Santa Cruz, sc22760; IF, 1:1,000); Conjugated ubiquitin (FK2; Enzo Life Sciences, BML-PW8800; IF, 1:10,000); RAP80 (Novus Biologicals, NBP1-87156; IF, 1:300); γ-H2AX (Millipore, 05–636, IF, 1:1000; WB, 1:500); Flag (WAKO, 018–22381; WB, 1:5000, Medical & Biological Laboratories Co., Ltd. (MBL), PM020; WB, 1:2000, IP, 1:200, M185-3L (FLA-1); IF and PLA, 1:5000); MDC1 (Abcam, ab11171; IF and PLA, 1:2,000, WB, 1:5000, IP, 1:300,); EHMT1 (MBL, D220-3; WB, 1:1000); EHMT2 (Cell signaling, 3306; WB, 1:500); histone H3 (Abcam, ab1791; WB: 1:5,000); H3K9me2 (Abcam, ab1220; WB: 1:1,000); HA (MBL, 561; WB, 1:2000, IP, 1:500); ATM (Santa Cruz, sc23921; WB, 1:200); MRE11 (Abcam, ab214; WB 1:500); NBS1 (GeneTex, GTX70224; WB 1:2000); H2A (Millipore, 07–146; WB, 1:1000); phospholyrated ATM (Ser1981) (Abcam, ab81292; WB, 1:5000, Cell signaling, 4526; IF and PLA, 1:500); TRF1 (GeneTex, GTX10579; PLA, 1:200). To generate a methyl-specific antibody against dimethyl-K45 of MDC1, dimethyl-K45 peptide NH2-C + AHGPE(Lys[Me]2)DFPLH-COOH was conjugated with keyhole-limpet hemocyanin and used to immunize rabbits. The antibody was generated from antiserum and the titres were determined by enzyme-linked immunosorbent assay (ELISA), using methylated and non-methylated peptides as antigens. The peptides and antibody were prepared by Operon Biotechnologies K.K. Tokyo. The antibody specificity toward methylated lysine was confirmed by immunoblotting, using cell lysates expressing the wild type or the K45A mutant of MDC1 (Fig. 3b).

### Immunofluorescence analysis and *in situ* PLA

Cells grown on grid glass coverslips were fixed in 4% formaldehyde for 10 min and permeabilized in 0.2% Triton X-100/PBS for 5 min at room temperature. Primary antibody with appropriate dilution was added to cells and incubated at 4 °C overnight followed by secondary antibodies coupled to Alexa 488 or 594 (Thermo Fisher Scientific) for 1 hr at room temperature. Coverslips were mounted onto glass slides (Matsunami) with FluorSAVE mounting medium (Millipore). PLA was performed following the manufacturer’s instructions using the Duolink anti-Mouse MINUS and anti-Rabbit PLUS *In Situ* PLA probes and the Duolink *In Situ* Detection Reagents Green (Olink Bioscience).

Images were analysed with a fluorescence microscope, Axio imagerA1 equipped with Plan-NEOFLUAR 63x/1.25 NA objective, the AxioCam MRc5 digital CCD camera and AxioVision software (Carl Zeiss Inc.), or Eclipse Ti-E (Nikon) equipped with Plan-APO 40x/0.95 NA objective, an ORCA-Flash 4.0 V2 camera (Hamamatsu Photonics) and the NIS Elements software (Nikon).

### Western Blot and Densitometry analyses

Samples were prepared by boiling in Laemmli sample buffer, separated by SDS-PAGE and transferred to PVDF membranes. Membranes were probed with indicated antibodies and signals were detected using enhanced chemiluminescence and LAS-3000/4000 mini (GE healthcare). Densitometric quantification was performed with ImageJ (National Institutes of Health) software.

### Immunoprecipitation assay

Nuclei were collected using swelling solution (5 mM PIPES, pH8.0, 85 mM KCl, 0.5% NP40), and nuclear chromatin-associated complexes were isolated using nuclear complex co-IP kit (Active motif #5400) according to the manufacturer’s protocols. Lysates were incubated with 30 μl Dynabeads Protein G (Thermo Fisher Scientific) and the indicated antibody complex for 4 hr at 4 °C. Beads were washed 5 times with TNE buffer (0.1% NP40, 50 mM Tris-HCl, pH 7.6, 150 mM NaCl, 5 mM EDTA). Thirty μl of Laemmli sample buffer was added to the beads to elute with heating at 96 °C for 5 min.

## Electronic supplementary material

Supplementary information
