# Supplementary material for: MDC1 methylation mediated by lysine methyltransferases EHMT1 and EHMT2 regulates active ATM accumulation flanking DNA damage sites
Source: Sci Rep. 2018 Jul 18;8:10888. doi: 10.1038/s41598-018-29239-3 (PMC6052104; doi:10.1038/s41598-018-29239-3)
Supplement: Supplementary file 1 — Supplementary information [file 41598_2018_29239_MOESM1_ESM.pdf]

SUPPLEMENTARY INFORMATION

MDC1 methylation mediated by lysine methyltransferases EHMT1 and EHMT2 regulates active ATM accumulation flanking DNA damage sites

Sugiko Watanabe, Makoto Iimori, David Virya Chan, Eiji Hara, Hiroyuki Kitao and Yoshihiko Maehara

Supplementary Figure legends

Supplementary Figure 1. SETD8, EHMT1 and EHMT2 are required for accumulation of ubiquitin conjugates and 53BP1 at DNA damage sites. (a and b) Immunofluorescence analysis of U2OS cells transfected with indicated siRNA oligonucleotides, and co-immunostained with indicated antibodies at 2 h after exposure to neocarzinostatin (NCS, 50 ng/ml for 15min). Knockdown efficiency of KMTs was confirmed by realtime RT-PCR (more than 85% and 70%, for KMTs in **a** and MLL4, and for MLL1-3, respectively). Scale bar, 10 μm.

Supplementary Figure 2. Depletion of SETD8 perturbed the distribution of MDC1 (a and b) Immunofluorescence analysis of U2OS cells transfected with (**a**) control, *SETD8*-, *EHMT1*- or *EHMT2*-targeting siRNAs, and (**b)** control or three *SETD8*-targeting siRNAs (*SETD8* siRNA-1 siRNA-2 and siRNA-3; see methods), and co-immunostained with indicated antibodies at 2 h after exposure to neocarzinostatin (NCS, 50 ng/ml for 15min). Scale bar, 10 μm.

Supplementary Figure 3. Knockdown of EHMT1 and EHMT2 impaired accumulation of ubiquitin conjugates, 53BP1 and RAP80 at DNA damage sites. (a - c) Immunoblotting analysis of whole-cell extracts from U2OS **(a)**, MCF7 **(b)** and TIG-3 **(c)** cells transfected with control, *EHMT1*-targeting siRNAs (*EHMT1* siRNA-1 and siRNA-2) or *EHMT2*-targeting siRNAs (*EHMT1* siRNA-1 siRNA-2 and siRNA-3; see methods), using the indicated antibodies. **(d and e)** Immunofluorescence analysis of U2OS **(d)** and TIG-3 **(e)** cells transfected with indicated siRNA oligonucleotides, and co-immunostained with indicated antibodies at 2 h after exposure to neocarzinostatin (NCS, 50 ng/ml for 15min).

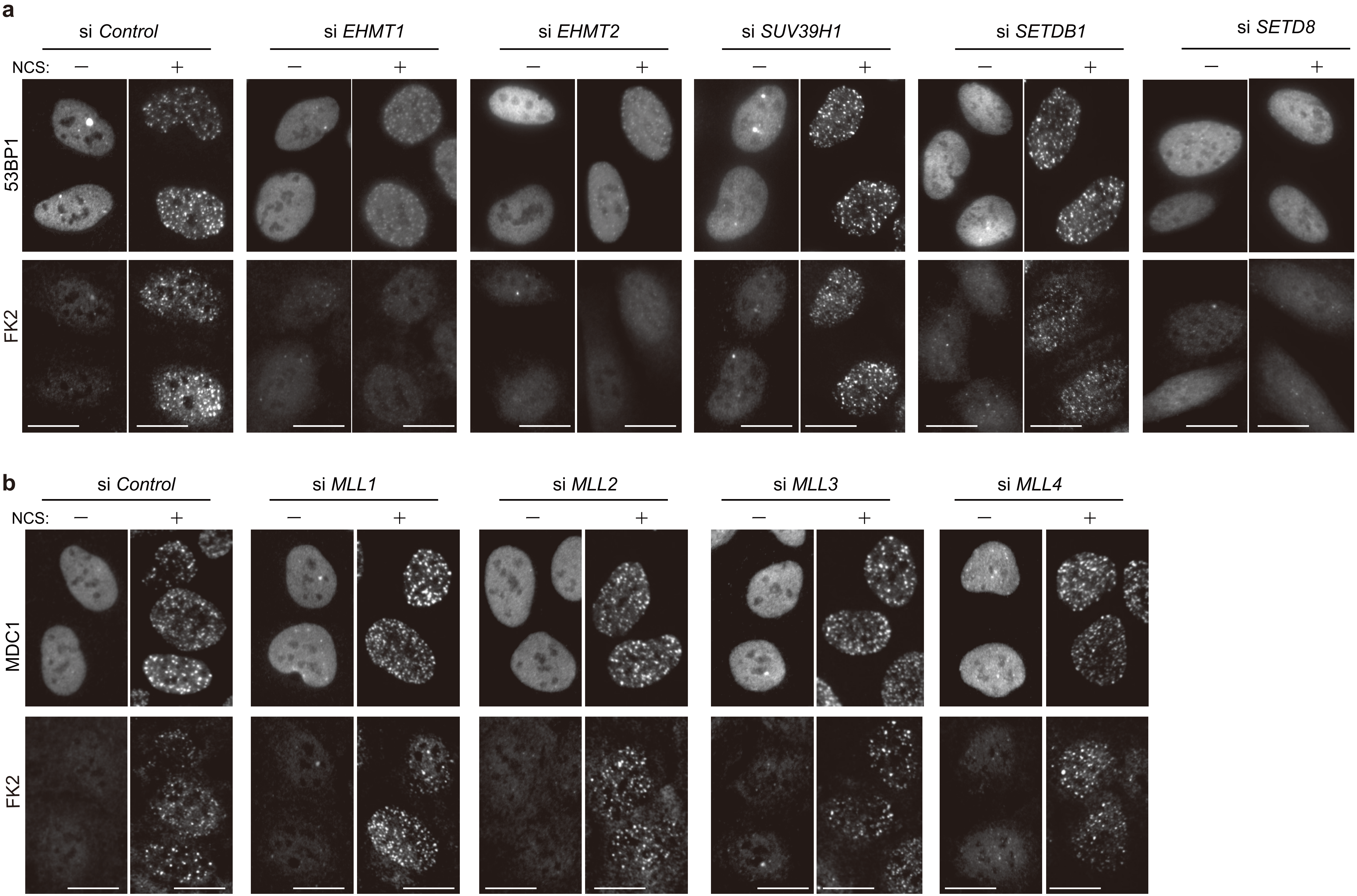

Supplementary Fig. 1 Watanabe et al.

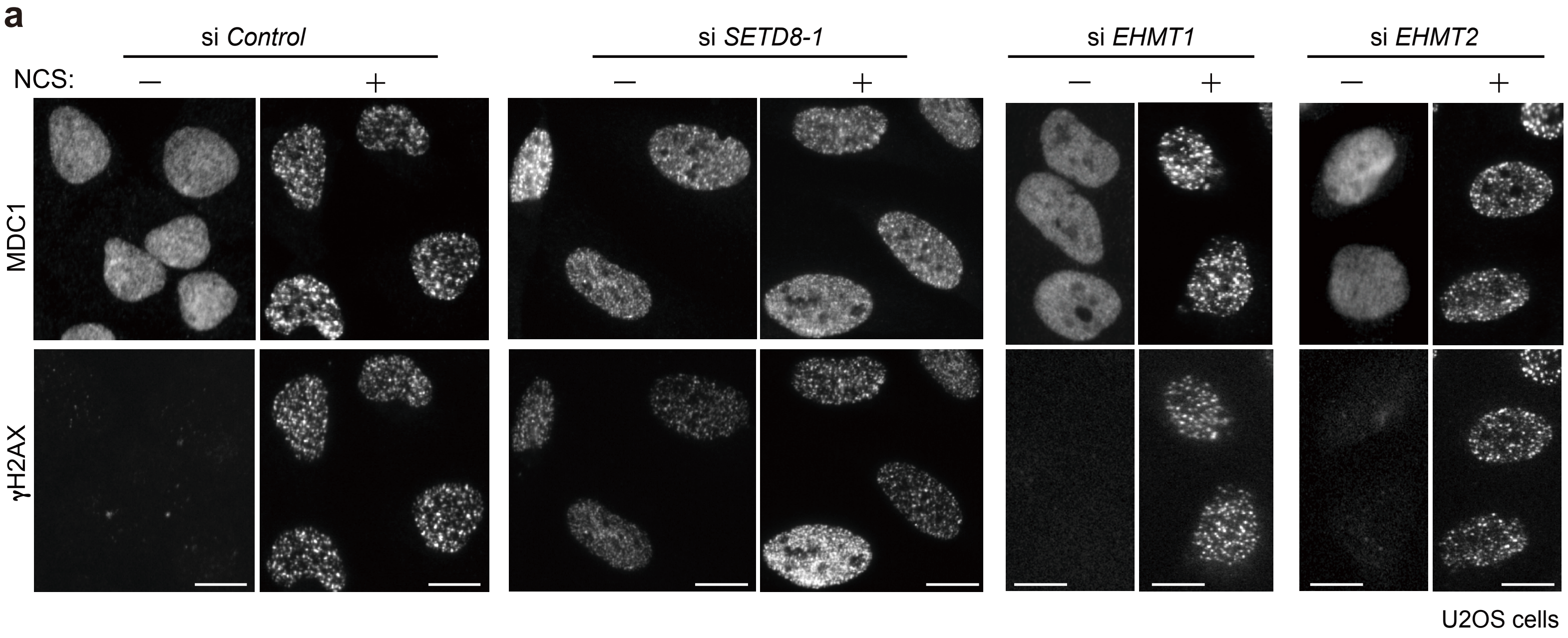

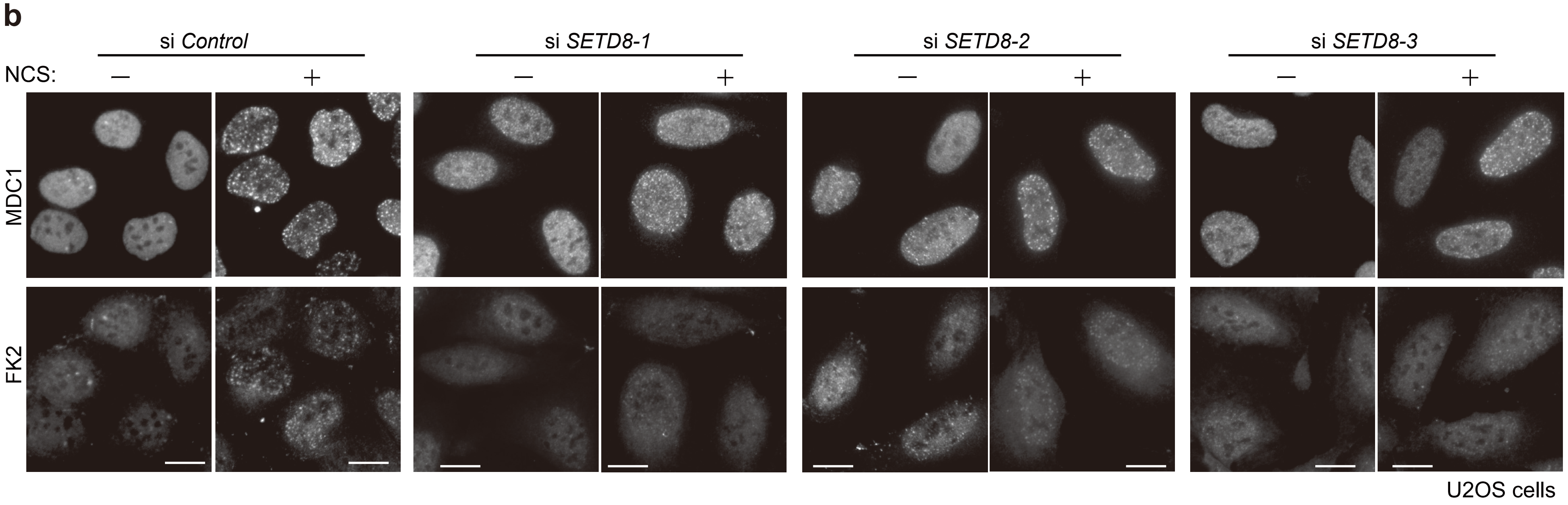

Supplementary Fig. 2 Watanabe et al.

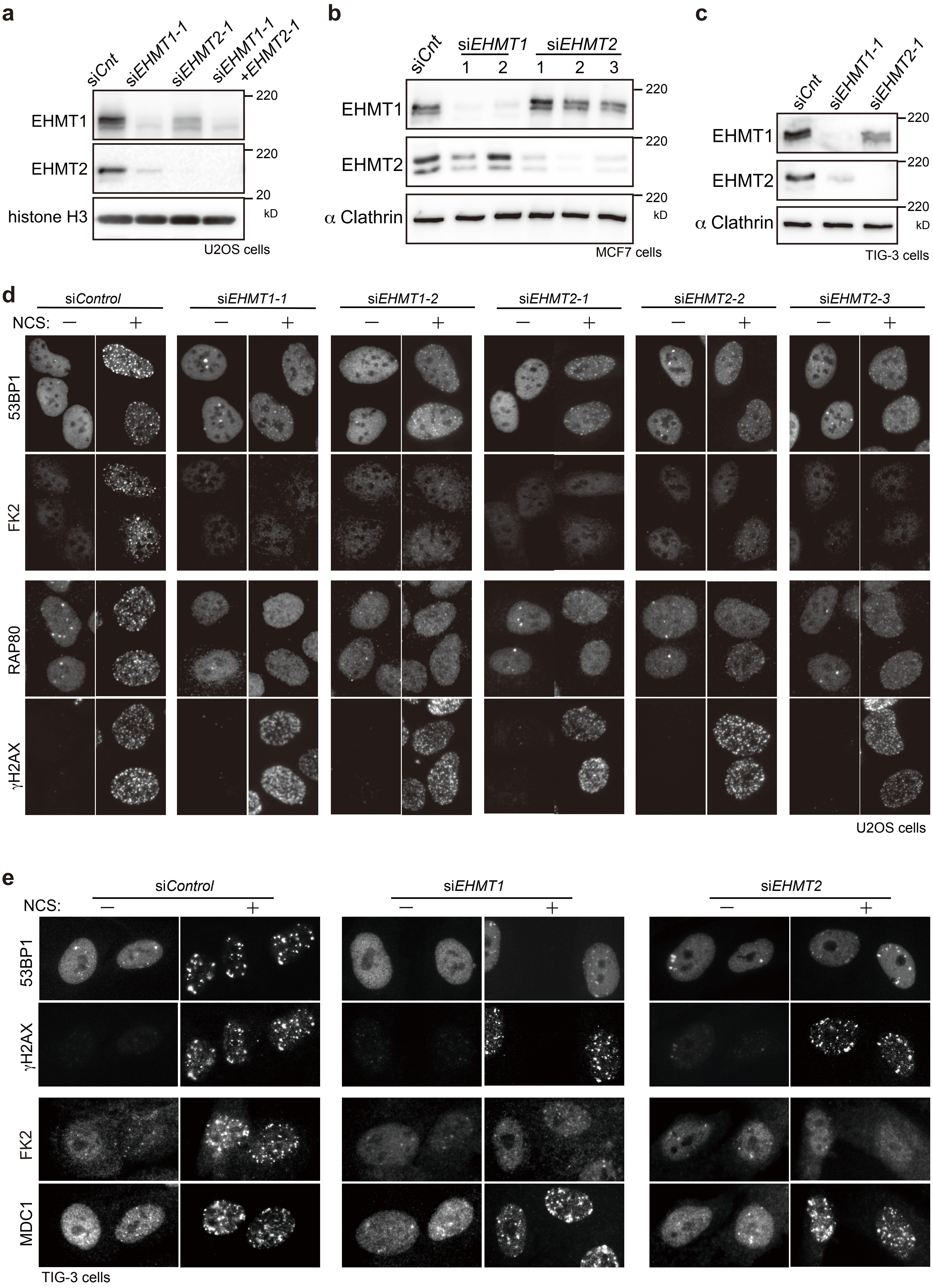

Watanabe et al. Supplementary Fig. 3
